# Clinico-characteristics of patients which correlated with preferable treatment outcomes in immunotherapy for advanced hepatocellular carcinoma: a systematic review and meta-analysis

**DOI:** 10.1097/JS9.0000000000000652

**Published:** 2023-08-17

**Authors:** Yani Wang, Wanyee Lau, Yafei Li, Yichen Tian, Yongrong Lei, Jianhua Wang, Feng Xia

**Affiliations:** a Key Laboratory of Hepatobiliary and Pancreatic Surgery, Institute of Hepatobiliary Surgery, Southwest Hospital, the First Hospital Affiliated to AMU (Southwest Hospital); b Key Laboratory of Biorheological Science and Technology, Ministry of Education, College of Bioengineering, Chongqing University; c Department of Epidemiology, College of Preventive Medicine, Army Medical University (Third Military Medical University), Chongqing; d Faculty of Medicine, Chinese University of Hong Kong, Prince of Wales Hospital, Shatin, New Territories, Hong Kong, SAR, People’s Republic of China

**Keywords:** clinico-pathological characteristic, hepatocellular carcinoma, immunotherapy, meta-analysis, systematic review

## Abstract

**Background and aims:**

Hepatocellular carcinoma (HCC) is the third-most lethal malignant tumor worldwide. The rapid development of immunotherapy utilizing immune checkpoint inhibitors for advanced HCC patients has been witnessed in recent years, along with numerous randomized clinical trials demonstrating the survival benefits for these individuals. This systematic review and meta-analysis aimed to identify specific clinico-pathological characteristics of advanced HCC patients that may lead to preferable responses to immunotherapy in terms of overall survival (OS), progression-free survival (PFS), and objective response rate (ORR).

**Methods:**

The included clinical trials were retrieved from PubMed, Embase, the Cochrane library, and the Web of Science databases published in English between 1 January 2002 and 20 October 2022. A systematic review and meta-analysis for first-line and second-line phase II/III studies were conducted on immunotherapy for patients with advanced HCC by using OS as the primary outcome measure, and PFS and ORR as the secondary outcome measures to obtain clinico-pathological characteristics of patients which might be preferable responses to programmed death-1 (PD-1) and programmed cell death-Ligand 1 (PD-L1) inhibitors. Toxicity and specific treatment-related adverse events (TRAEs) were also determined.

**Results:**

After screening 1392 relevant studies, 12 studies were included in this systematic review and meta-analysis to include 5948 patients. Based on the analysis of interaction, the difference in OS after first-line immunotherapy between the subgroups of viral hepatitis [hazard ratio (HR)=0.73 vs 0.87, *P* for interaction=0.02] and macrovascular invasion and/or extrahepatic spread (HR=0.73 vs 0.89, *P* for interaction=0.02) were significant. The difference in PFS between the subgroups of viral hepatitis was highly significant (pooled HR=0.55 vs 0.81, *P* for interaction=0.007). After second-line immunotherapy, the difference in ORR between the subgroups of Barcelona Clinic Liver Cancer was significant (pooled ES=0.12 vs 0.23, *P* for interaction=0.04). Compared with PD-L1 inhibitors, PD-1 inhibitors may have a higher probability to cause TRAEs. Diarrhea, increased aspartate aminotransferase, and hypertension were the top three TRAEs.

**Conclusions:**

This systematic review and meta-analysis represents the first pilot study aimed at identifying crucial clinico-pathological characteristics of patients with advanced HCC that may predict favorable treatment outcomes in terms of OS, PFS, and ORR to immunotherapy. Findings suggest that patients with viral hepatitis positivity (especially hepatitis B virus) and macrovascular invasion and/or extrahepatic spread may benefit more in OS when treated with PD-1/PD-L1 immune checkpoint inhibitors.

## Introduction

Highlights

- A meta-analysis about the relationship between clinico-pathological features and treatment responses to immunotherapy.
- Patients with viral hepatitis positivity (especially hepatitis B virus) and with macrovascular invasion and/or extrahepatic spread may benefit more in overall survival to first-line immunotherapy.
- Programmed death-1 inhibitors may have a higher probability to cause treatment-related adverse events compared to programmed cell death-Ligand 1 inhibitors.

Hepatocellular carcinoma (HCC) is a histological subtype of primary liver cancer, which accounts for 90% of all primary liver cancers. It is the third-most common cause of cancer-related mortality in the world^1,2^. Systemic therapy, including immunotherapy, molecular targeted therapy, and chemotherapy, is the mainstay of treatment for patients with advanced HCC or with noncurable recurrence after surgery^3^. Cancer immunotherapy has recently revolutionized treatment of solid malignancies. The use of a checkpoint inhibitor is a novel and effective way to treat many cancers based on the principle of stimulating patient’s own immune system to strive against cancer^4^. Nowadays, studies on the checkpoint programmed death-1 (PD-1) and programmed cell death-Ligand 1 (PD-L1) inhibitors have become a principal focus of current HCC immunotherapy research^5^. The results of the IMbrave-150 trial have led to many international guidelines to recommend the combination of the checkpoint inhibitor Atezolizumab with the VEGF antibody Bevacizumab to be the first-line treatment for patients with advanced HCC^6,7^. Furthermore, when compared to Sorafenib, recent studies showed that Durvalumab plus Tremelimumab to achieve superior overall survival (OS), and Atezolizumab plus Cabozantinib to yield superior progression-free survival (PFS)^8^.

However, it is acknowledged that not all patients can have effective treatment outcomes to immunotherapy, despite the promising prognostic results obtained from immunotherapy on many patients with advanced HCC^9^, and up to one fourth of all treated patients developed high-grade immune-related adverse events^5^. A major challenge now is to be able to identify patients who are likely to benefit from checkpoint inhibitor treatment. Little is known about the potential use of biomarkers or clinico-pathologic characteristics of HCC patients in predicting response to immunotherapy and to guide medical practitioners in clinical decision-making. Previous studies have demonstrated that specific laboratory parameters like alpha-fetoprotein (AFP) and its changes to treatment, inflammatory parameters like C-reactive protein, and etiological factors of HCC like hepatitis B or C have strong correlations with patient response to immunotherapy^10–12^.

Although many clinical trials have been reported on immunotherapy using PD-1 and PD-L1 inhibitors to treat HCC, to our knowledge, the relationship between treatment outcomes of immunotherapy for HCC with clinico-pathological characteristics of patients has not been evaluated systematically. This systematic review and meta-analysis on contemporary studies was conducted to assess the relationship between clinico-pathological features of patients with advanced HCC with treatment responses to PD-1 and PD-L1 inhibitors.

## Methods

This systematic review and meta-analysis focused on clinico-characteristics of patients which correlated with preferable treatment outcomes in immunotherapy for advanced HCC. The work has been reported in line with PRISMA^13^ (Preferred Reporting Items for Systematic Reviews and Meta-Analyses) (Supplemental Digital Content 1, http://links.lww.com/JS9/A901), (Supplemental Digital Content 2, http://links.lww.com/JS9/A902) and AMSTAR^14^ (Assessing the methodological quality of systematic reviews) Guidelines (Supplemental Digital Content 3, http://links.lww.com/JS9/A903).

### Search strategy

The databases of PubMed (included in MEDLINE), Cochrane Library, and Web of Science were reached using the keywords liver neoplasms, HCC, liver cancer, monoclonal antibody, PD-1 and PD-L1 immune checkpoint inhibitors (ICIs), immunotherapy, and clinical trial to identify and to select relevant articles published from 1 January 2002 to 20 October 2022. The European Society for Medical Oncology (ESMO) and American Society of Clinical Oncology (ASCO) Meetings’ memoir in 2022 were screened for more recently conducted randomized clinical trials (RCTs). The comprehensive searching strategies were shown in Table S1 (Supplemental Digital Content 4, http://links.lww.com/JS9/A904).

### Criteria for selecting trials

**Figure 1:**
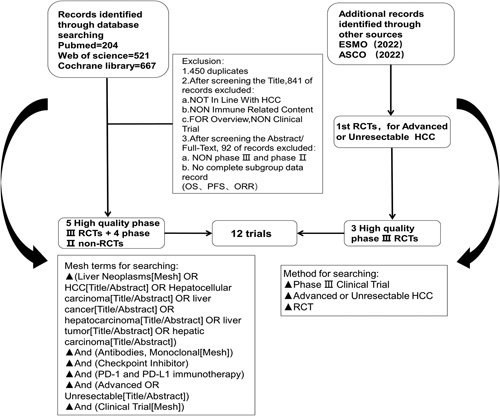
Flowchart of study selection. The description of specific screening and elimination methods. Twelve studies were included finally. HCC, hepatocellular carcinoma; ESMO, European society for medical oncology; ASCO, American society of clinical oncology; RCT, randomized controlled trial; OS, overall survival; PFS, progression-free survival; ORR, objective response rate.

The selection criteria were: phase III and phase II clinical trials on patients with advanced HCC; treatment using immunosuppressive agents that inhibit PD-1 and PD-L1 signaling pathways; trials with at least one endpoint to include OS, PFS, and objective response rate (ORR) being clearly stated in the form of subgroups. The exclusion criteria were: studies not on immunotherapy for patients with advanced HCC; studies in which end points of interest (OS, PFS, or ORR) were not reported or no complete subgroup data record was provided. The specific inclusion and exclusion criteria are shown in Figure 1.

### Quality assessment and data extraction

The risk of bias in RCTs for quality assessment was assessed by the Cochrane Collaboration’s tool^15^ using six fields and seven evaluation items. The risk was judged to be ʻlow bias, unclear, high biasʼ. The quality of RCTs was scored by the Jadad scale. Non-RCTs were marked by the Minors scale^16^. The final quality assessment results are shown in Supplementary Figure 6 (Supplemental Digital Content 4, http://links.lww.com/JS9/A904) as well as Supplementary Tables S2 (Supplemental Digital Content 4, http://links.lww.com/JS9/A904) and S3 (Supplemental Digital Content 4, http://links.lww.com/JS9/A904). RCT articles with a score of greater than or equal to 5 (full score=7) and non-RCTs with a score of greater than or equal to 13 (full score=16, without a control group) were considered as high-quality. The work was jointly completed by WYN and TYC, and the ultimate outcome was judged by LYR. To better appraise the degree of clinical benefit of the included clinical trials, eight RCTs of first-line treatment were graded using the ESMO-MCBS scoring. Studies were excluded with a score of less than or equal to 2.0^17,18^. The remaining studies were analyzed by merging and comparing treatment outcomes by subgroup analysis. The methodological quality of this article was evaluated using the AMSTAR checklist^14^ (Supplemental Digital Content 3, http://links.lww.com/JS9/A903).

Study’ features, patients’ characteristics, and final treatment results collected included the following: publication year, first author, trial type, competing interests, treatment arms (plus control arms), subject inclusion criteria, age, sex, number and size of tumors, median follow-up, adverse events, and primary end points. In this study, the primary outcome was the hazard ratio (HR) of OS. PFS and ORR were used as secondary outcome measures.

### Statistical analysis

The HR of OS and PFS of different clinico-pathological characteristics (including age, sex, and AFP) in each of the included studies were extracted and pooled into subgroups to obtain their corresponding values, 95% CI and *P*-values were determined by utilizing RevMan 5.4 (For viral hepatitis, fixed-effect inverse-variance meta-analysis was first used to combine the HR values of (hepatitis B virus) HBV and (hepatitis C virus) HCV in each RCT, then merging was performed among the RCTs. In addition, separate comparisons were made between positive HBV and nonhepatitis, as well as between positive HCV and nonhepatitis). If the HR of OS or PFS was not recorded in the form of subgroups in the studies, meta-analysis was performed to obtain the effect sizes of ORR using Stata 17 (also for subgroups). For evaluation of heterogeneity, the *χ* ^2^-test and inconsistency *I* ^2^ were applied. A large *χ* ^2^ represents a great deviation. When a low heterogeneity as represented by *I* ^2^<50%, the fixed-effect model was chosen. When *I* ^2^>50%, the random effect model was selected. Publication bias was evaluated graphically using the funnel chart and examined with the Egger’s test. After a case-by-case elimination and changing in the merge model (conversion between the random effects model and fixed effects model) to evaluate sensitivity to verify stability of the analysis, the estimated HR and OR values (95% CI, *P*-values), were compared, and the clinico-pathological characteristics of patients which were associated with preferable treatment outcomes to immunotherapy in each of the subgroups were determined, and interaction analyses were employed to identify subgroups with significant differences. The arithmetic average method and interaction analyses were used to calculate the incidences of adverse events.

### Aims of the study

The main aim of this study was to comprehensively and systematically review and to analyze high-quality RCTs published from 2002 to 2022 on first-line immunotherapy and the related second-line immunotherapy studies on patients with advanced HCC. This meta-analysis focused on OS of patients, supplemented by PFS and ORR. The relationship between clinico-pathological features with preferable treatment outcomes using PD-1 and PD-L1 inhibitors was determined to identify key characteristics that were significantly associated with treatment of immunotherapy on the OS, PFS, and ORR. The toxicity and specific treatment-related adverse events (TRAEs) of ICIs were also determined.

## Results

### Characteristics of the included studies

Searching from the databases, 1392 studies were found between January 2002 and October 2022 using the keywords of our search, and 101 studies were identified after preliminary evaluation as potentially related clinical trials. After excluding the first-line nonphase III studies, five high-quality studies were identified. To increase reliability in ORR of first-line treatment, a large sample size phase II non-RCT study (RESUC^19^) was also included. Furthermore, by searching the latest research published at the international conference of EMSO and ASCO, a total of eight high-quality RCTs and one non-RCT study were finally included (Table 1A). The funnel plot for first-line phase III studies shows a slight asymmetry (Supplementary Figure 1, Supplemental Digital Content 4, http://links.lww.com/JS9/A904), with COSMIC-312 outside of the CI^20^. However, the Egger’s test showed *P*=0.068 (*P*>0.05) indicating there was no obvious publication bias. There were only a few second-line studies that recorded OS and PFS in the form of subgroups, and only four second-line studies that recorded ORR that could be included in this study for further analysis (Table 1B).

**Table 1:** First-line and second-line immunotherapy-characteristics of studies included in meta-analysis.

									OS		PFS		
**A First-line** trial	Test result	n	Primary end point	Median follow-up mo	Jadad score	Phase	Treatment arms	Control arms	Median mo	HR (95% CI)	Median mo	HR (95% CI)	0RR %
COSMIC-312 2022	Negative	649	OS PFS	13.3 (10.5–16)	7	III	Atezolizumab +Cabozantinib	Sorafenib	15.4/15.5	0.90 (0.69–1.18)	6.8/4.2	0.63 (0.44–0.91)	11
IMbrave-150 2021	Positive	501	OS PFS	17.6 (0.1–28.6) /10.4 (0–27.9)	7	III	Atezolizumab +Bevacizumab	Sorafenib	19.2/13.4	0.66 (0.52–0.85)	6.9/4.3	0.65 (0.53–0.81)	30
ORIENT-32 2021	Positive	571	OS PFS	10 (8.5–11.7) /10 (8.4–11.7)	7	III	Sintilimab +Bevacizumab	Sorafenib	Na/10.4	0.57 (0.43–0.75)	4.6/2.8	0.56 (0.46–0.70)	21
CheckMate-459 2021	Negative	743	OS	15.2 (5.7–28) /13.4 (5.7–25.9)	6	III	Nivolumab	Sorafenib	16.4/14.7	0·85 (0.72–1.02)	3.7/3.8	0.93 (0.79–1.10)	15
HIMALAYA 2022	Positive	782	OS	33.18 (31.74–34.53)/ 32.23 (30.42–33.71)	5	III	Tremelimumab +Durvalumab	Sorafenib	16.43/13.77	0.78 (0.65–0.93)	3.78/4.07	0.90 (0.77–1.05)	20.1
LEAP-002 2022	Negative	794	OS PFS	32.1	7	III	Pembrolizumab +Lenvatinib	Lenvatinib +Placebo	21.2/19.0	0.840 (0.708–0.997)	8.2/8.0	0.867 (0.734–1.024)	26.1
RATIONALE -301 2022	Positive	674	OS	≥33	7	III	Tislelizumab	Sorafenib	15.9/14.1	0.85 (0.712–1.019)	2.1/3.4	1.11 (0.92–1.33)	14.3
SHR-1210-III -310 2022	Positive	543	OS PFS	—	7	III	Camrelizumab +Rivoceranib	Sorafenib	22.1/15.2	0.62 (0.49–0.80)	5.6/3.7	0.52 (0.41–0.65)	25.4
RESCUE 2021	—	70	ORR	16.7(11.1-18.2)	—	III	Camrelizumab	—	—	—	5.7	—	34.3
B Second-line Trial	n	Median follow-up mo	Minors score	Treatment Arms	OS Median, mo	PFS Median, mo	TTP Median, mo	ORR, % (95% CI)	DCR, % (95% CI)				
KEYNOTE-224 2018	104	~24	14	Pembrolizumab	13.2 (9.7–15.3)	4.9 (3.5–6.7)	4.8 (3.9–7.0)	18.3 (11.4–27.1)	61.5				
CheckMate-040 2020	148	30.7 (29.9–34.7)	15	Nivolumab +Ipilimumab	22.8 (9.4–Na)	—	—	32(20-47)	54				
RESCUE 2021	120	14.0 (9.6–17.0)	14	Camrelizumab +Apatinib	—	5.5 (3.7–5.6)	—	22.5 (15.4–31.0)	75.8 (67.2–83.2)				
RATIONALE-208 2022	249	12.7 (0.1–37.0)	13	Tislelizumab	13.2 (10.8–15.2)	2.7 (1.4–2.8)	—	13 (9–18)	53 (47–59)				

Jadad Score, a tool to mark the literature quality of RCTs. Minors Score, a tool to mark the literature quality of non-RCTs. DCR, disease control rate; HR, hazard ratio; mo, month; Na, not available; ORR, objective response rate; OS, overall survival; PFS, progression-free survival; TTP, time to Progression.

Of the eight RCT studies on first-line treatment, five had a positive outcome, and the remaining three had a negative outcome. Of the five studies with an adequate length of median follow-up, and when compared with the control group, SHR-1210-III-310 showed the longest prolongation in a median OS of 6.9 months with first-line treatment (HR=0.62, 95% CI: 0.49–0.80)^21^. COSMIC-312 and IMbrave-150 tied for the first place in prolonging a median PFS of 2.6 months (HR=0.63, 95% CI: 0.44–0.91; HR=0.65, 95% CI: 0.53–0.81)^20,22^. The ORR of the combined treatment using Atezolizumab and Bevacizumab in IMbrave-150 was 30%, ranking at the top above all the remaining seven studies which used either Sorafenib or Lenvatinib as the control groups (Table 1A). There were two studies which used either Nivolumab or Tislelizumab as a single drug, five studies which used immunotherapy plus targeted therapy (Atezolizumab + Cabozantinib, Pembrolizumab + Lenvatinib, Camrelizumab + Rivoceranib, Sintilimab + Bevacizumab, Atezolizumab + Bevacizumab, respectively), and the remaining study used double immunotherapy (Tremelimumab + Durvalumab). In all these studies, OS and PFS were the most common primary end points used. In CheckMate-459^23^, HIMALAYA^8^, RATIONALE-301^24^, OS was the only primary end point used.

For second-line studies, KEYNOTE-224 reported the long-term efficacy and safety of immunotherapy with 2.5 years of follow-up^25^. RATIONALE-208 studied the treatment outcomes of Tislelizumab as a second-line immunotherapy^26^. RATIONALE-301 demonstrated Tislelizumab to be effective as a first-line treatment, thus confirming the versatility of its use of Tislelizumab in immunotherapy of advanced HCC. As RESCUE included both first-line and second-line treatments, it was also included to be analyzed as a second-line study. Of the four non-RCTs, CheckMate-040 ranked first in achieving the best OS and ORR (median 22.8 months, 32%)^27^, and RESCUE achieved the best treatment outcome in PFS with a median of 5.5 months (Table 1B).

### Subgroup analysis of first-line immunotherapy to correlate clinico-pathological characteristics with OS

First-line immunotherapy for advanced HCC has developed rapidly in recent years, and most published phase III clinical studies were reported in 2022. Sorafenib was used as the control group in seven of the eight studies. CheckMate-459 was the first report but it failed to reach the significance level as defined in the study protocol. The subsequent success of IMbrave-150 by using Atezolizumab–Bevacizumab revolutionized immunotherapy in patients with unresectable or metastatic HCC.

**Figure 2:**
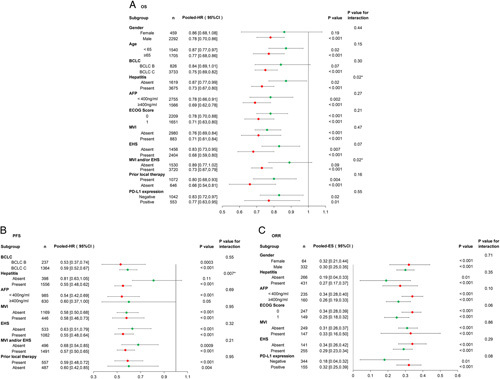
Forest plot analysis of each subgroup to first-line immunotherapy. (A) OS. Viral hepatitis and MVI and/or EHS subgroups had significant difference in OS according to interaction analysis. (B) PFS. Viral hepatitis had significant difference in PFS according to interaction analysis. (C) ORR. In each subgroup, if *I* ^2^ greater than 50%, the random effects model was used, on the contrary, the fixed effects model was used. *P*<0.05 was defined as statistically significant. *P*-value for interaction indicated whether there were significant differences in subgroups, and *P*<0.05 was defined as significant difference. Prior local therapy refers to surgery, ablation, liver transplant, radiotherapy and so on. OS, overall survival; BCLC, Barcelona Clinic Liver Cancer; AFP, alpha-fetoprotein; ECOG, Eastern Cooperative Oncology Group; MVI, macrovascular invasion; EHS, extrahepatic spread. PD-L1, immune checkpoint inhibitors, programmed death-ligand 1.

Eight studies, which included 5257 patients reported OS in detail in the subgroups of patients with various clinico-pathological characteristics, except for COSMIC-312 which only reported on etiology and whether there was macrovascular invasion (MVI) or extrahepatic spread (EHS). At the numerical level using pooled HR on subgroup analysis and sensitivity analysis (detailed in the Supplementary Material, Supplemental Digital Content 4, http://links.lww.com/JS9/A904), meta-analysis showed the clinico-pathological characteristics of patients with advanced HCC which responded with preferable OS to immunotherapy were no prior local therapy, viral hepatitis positivity , with EHS, AFP ≥400 ng/ml, Eastern Cooperative Oncology Group (ECOG) 1, with MVI, with MVI and/or EHS, Barcelona Clinic Liver Cancer (BCLC) C disease, age greater than or equal to 65 years, and male (Fig. 2A).

**Figure 3:**
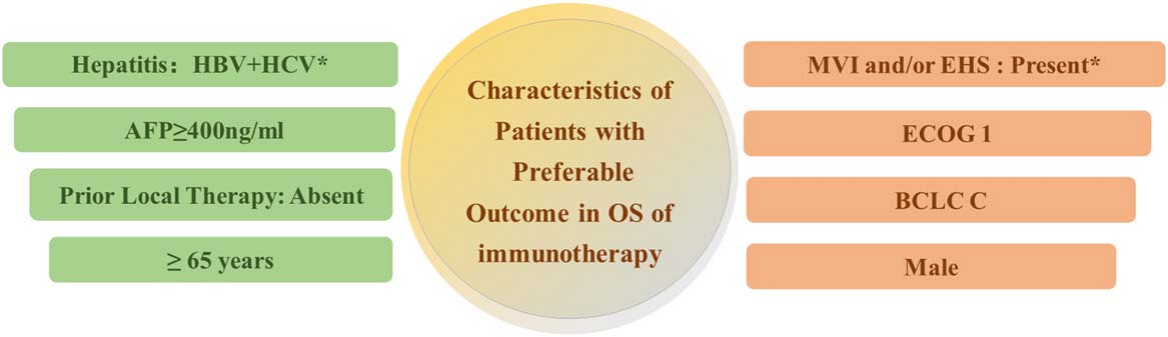
Clinico-pathological characteristics of patients with preferable outcome in OS of immunotherapy. Clinico-pathological characteristics with × were that characteristics showed significant correlation in the subgroups as determined by interaction effect. The remaining clinico-pathological characteristics in the figure showed improved responses in each subgroup based on pooled HR values. Prior local therapy refers to surgery, ablation, liver transplant, radiotherapy and so on. EHS, extrahepatic spread; AFP, alpha-fetoprotein; ECOG, Eastern Cooperative Oncology Group; MVI, macrovascular invasion; BCLC, Barcelona Clinic Liver Cancer; PD-L1, immune checkpoint inhibitors, programmed death-ligand 1.

The results of pooled HR on these clinico-pathological characteristics which were associated with preferable OS to immunotherapy are shown in Figure 3. The significant differences in OS were obtained with the clinico-pathological characteristics based on interaction analysis were with and without viral hepatitis (HR=0.73 vs 0.87, *P* for interaction=0.02) and with and without MVI and/or EHS (HR=0.73 vs 0.89, *P* for interaction=0.02). For viral hepatitis, the interaction analysis revealed significant difference in OS was obtained when considering the patients with HBV infection and those without non-hepatitis (HR=0.70 vs 0.87, *P* for interaction=0.008) (Supplementary Figure 2, Supplemental Digital Content 4, http://links.lww.com/JS9/A904). Other clinico-pathological characteristies were significantly associated with OS based on meta-analysis except for BCLC B disease (*P*=0.07), absent MVI and/or EHS (*P*=0.09) and female sex (*P*=0.19) (Fig. 2A).

To mitigate the potential impact of variations in specific studies conducted by different method, we excluded studies involving mono-immunotherapy and double immunotherapy from our analysis (CheckMate-459, RATIONALE-301, HIMALAYA). The resultant findings remain consistent with our initial results, thereby providing further validation of the stability and reliability of our study outcomes (Supplementary Figure 3, Supplemental Digital Content 4, http://links.lww.com/JS9/A904).

### Subgroup analysis of first-line immunotherapy using the ESMO scoring on OS

For any clinical trial, clinical benefits and costs are both important. The ESMO Clinical Benefit Scale (ESMO-MCBS (v1.1)) is a tool that can be used to assess the degree of clinical benefit. Based on this scale the included studies were scored (Supplementary Table 4, Supplemental Digital Content 4, http://links.lww.com/JS9/A904). Studies with a score of less than or equal to 2.0 were removed. Subgroup analysis was then conducted on the remaining studies.

The two single drug studies of CheckMate-459 (Nivolumab) and RATIONALE-301 (Tislelizumab) showed significantly lower toxicity when compared with Sorafenib. Meanwhile, ORIENT-32^28^, CheckMate-459, and HIMALAYA showed improved quality of life in patients, and these four studies gained their corresponding bonus points. As LEAP-002 with only 2.0 points was excluded^29^, the final forest plot was drawn on the remaining seven studies (Supplementary Figure 4, Supplemental Digital Content 4, http://links.lww.com/JS9/A904). The results on the numerical level of pooled HR showed consistency with the results obtained before the ESMO scoring. The difference in OS in the viral hepatitis subgroup remained significant (HR=0.72 vs 0.88, *P* for interaction=0.02), while it became just significant in the MVI and/or EHS subgroup (HR=0.72 vs 0.86, *P* for interaction=0.05).

### Subgroup analysis of first-line immunotherapy to correlate clinico-pathological characteristics with PFS

HR subgroup analysis was conducted on four studies (COSMIC-312, IMbrave-150, ORIENT-32, SHR-1210-III-310) with 2264 patients to correlate clinico-pathological characteristics with PFS. After meta-analysis, the subgroups that showed significant statistical differences were selected to construct the forest plot. Figure 2B, based on the numerical level, showed the clinico-pathological characteristics of patients which were significantly associated with preferable effectiveness on PFS after immunotherapy were patients in BCLC B, with viral hepatitis positivity, AFP less than 400 ng/ml, with EHS, with MVI and/or EHS, and received prior local therapy. These results were stable after sensitivity analysis (detailed in the Supplementary Material, Supplemental Digital Content 4, http://links.lww.com/JS9/A904). The difference in PFS between the subgroups of patients with and without viral hepatitis was highly significant (pooled HR=0.55 vs 0.81, *P* for interaction=0.007), especially in HBV subgroup, the interaction analysis revealed significant difference in PFS was obtained when considering the patients with HBV infection and those without non-hepatitis (HR=0.53 vs 0.81, *P* for interaction=0.004) (Supplementary Figure 2, Supplemental Digital Content 4, http://links.lww.com/JS9/A904). The results of the other subgroups of patients with different clinico-pathological characteristics were also statistically significant, with the exception of the parameter of hepatitis (*P*=0.11) and AFP greater than or equal to 400 ng/ml (*P*=0.05).

### Subgroup analysis of first-line immunotherapy to correlate clinico-pathological characteristics with ORR

Since most of the RCTs on first-line immunotherapy did not provide subgroup data on ORR, a non-RCT study (RESUCE) was added to include altogether three studies (IMbrave-150, CheckMate-459, RESCUE) with 1314 patients. As the included non-RCT study was a single arm study with no control group, the single group rate meta-analysis was used.

Figure 2C, based on numerical level, shows the clinico-pathological characteristics of patients which correlated with preferable ORR to immunotherapy were viral hepatitis positivity, AFP less than 400 ng/ml, ECOG 0, no EHS, and positive expression of PD-L1. However, the differences within each subgroup were not statistically significant.

### Subgroup analysis of second-line immunotherapy to correlate clinico-pathological characteristics with ORR

In the treatment of advanced HCC, drug resistance has always been a hard nut to crack^30^, and second-line pharmaceuticals are often required. In recent years, immunotherapy has developed swiftly as the second-line treatment of HCC^31^. To find out the clinico-pathological characteristics of patients who responded preferable to immunotherapy after developing drug resistance or ineffectiveness to first-line treatment, four non-RCTs were included in this meta-analysis on 621 patients (Table 1B).

**Figure 4:**
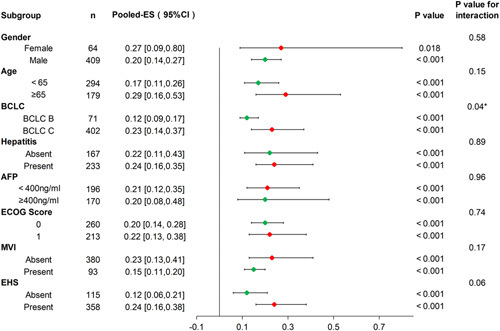
ORR-Forest plot analysis of each subgroup of second-line immunotherapy. In each subgroup, if *I* ^2^ greater than 50%, the random effects model was used, on the contrary, the fixed effects model was used. *P*<0.05 was defined as statistically significant. *P*-value for interaction indicated whether there were significant differences in subgroups, and *P*<0.05 was defined as significant difference (BCLC subgroup had significant difference in ORR). ORR, objective response rate; BCLC, Barcelona Clinic Liver Cancer; AFP, alpha-fetoprotein; ECOG, Eastern Cooperative Oncology Group; MVI, macrovascular invasion; EHS, extrahepatic spread.

These four studies provided concrete data on ORR, and KEYNOTE-224 had the longest follow-up. Figure 4, based on numerical level, shows the clinico-pathological characteristics of advanced HCC patients which correlated with preferable ORR to second-line immunotherapy were female, age greater than or equal to 65 years, BCLC C disease, with EHS, and no MVI (all *P*<0.05). The patients with BCLC C disease and BCLC B disease had significant difference in ORR to second-line treatment based on interaction analysis (pooled effect sizes=0.12 vs 0.23, *P* for interaction=0.04).

### Summing and ranking of adverse events

The use of ICIs in patients with advanced HCC, as with any antitumor drugs, is commonly accompanied with a variety of adverse events. By dividing the nine studies into two groups according to inhibition of the PD-1 pathway (the PD-1 group) or the PD-L1 pathway (the PD-L1 group), there were six studies in the PD-1 group and three studies in the PD-L1 group. Table 2 shows that the incidence rates of treatment-related all grade AEs and grade ≥3 TRAEs in the PD-L1 group were lower than that in the PD-1 group (86.1 vs 85.3%, 44.3 vs 42.3%, respectively). In addition, after interaction analyses, similar conclusions were reached (Supplementary Figure 5, Supplemental Digital Content 4, http://links.lww.com/JS9/A904). In particular, the incidence of grade ≥3 TRAEs of SHR-1210-III-310 in the PD-1 group was extremely high at 80.9%. On the other hand, the overall acute toxicity rate of CheckMate-459 was the lowest, followed closely by RATIONALE-301.

**Table 2:** Safety summary of the programmed death-1 group and the programmed cell death-Ligand 1 group.

	PD-1 group							PD-L1 group			
Safety, *n* (%)	ORIENT -32 (*n*=380)	CheckMate -459 (*n*=367)	LEAP -002 (*n*=395)	SHR-1210-III-310 (*n*=272)	RATIONALE -301 (*n*=338)	RESCUE (*n*=70)	Portion (%)	IMbrave -150 (*n*=329)	COSMIC -312 (*n*=429)	HIMALAYA (*n*=388)	Portion (%)
All-cause all grade AE Treatment-related all grade AE	376 (98.9)	—	—	—	325 (96.2)	—	97.6	322 (97.9)	—	378 (97.4)	97.6
	337 (88.7)	257 (70.0)	381 (96.5)	265 (97.4)	259 (76.6)	69 (98.6)	86.1	284 (86.3)	399 (93.0)	294 (75.8)	85.3
Grade≥3 AE Treatment-related Grade≥3 AE	207 (54.5)	—	—	—	163 (48.2)	—	51.5	230 (69.9)	324 (75.5)	196 (50.5)	65.4
	128 (33.7)	82 (22.3)	247 (62.5)	220 (80.9)	75 (22.2)	55 (78.6)	44.3	149 (45.3)	236 (55.0)	100 (25.8)	42.3
Serious AE^a^ Treatment-related serious AE	123 (32.4)	—	—	—	101 (29.9)	—	31.2	160 (48.6)	—	157 (40.5)	44.2
	65 (17.1)	43 (11.7)	—	66 (24.3)	40 (11.8)	23 (32.9)	16.6	76 (23.1)	78 (18.2)	68 (17.5)	19.4
AE leading to death	6 (1.6)	4 (1.1)	—	—	15 (4.4)	1 (1.4)	2.3	—	—	30 (7.7)	7.7
AE leading to dose interruption of any study treatment	188 (49.5)	—	—	—	37 (10.9)	—	31.3	195 (59.3)	352 (82.1)	53 (13.7)	52.4

a serious AE refers to events that endanger the safety of drug use of the people, such as pneumonitis, hepatitis, myocarditis, myasthenia gravis, etc. Data are *n* (%).AE, adverse event.

**Figure 5:**
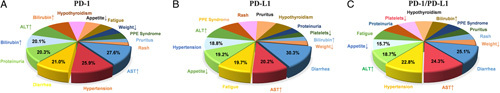
The pie chart of treatment-related adverse events for all grades. From left to right: (A) PD-1; (B) PD-L1; (C) PD-1; and PD-L1. The arithmatic average method was used to calculate the incidence of adverse events, and these sector charts were drawn based on the percentage of treatment-related adverse events. PPE syndrome, Palmar-plantar erythrodysesthesia; ALT, alanine aminotransferase; AST, aspartate aminotransferase; ↓ for decrease; ↑ for increase.

The frequent specific TRAEs rates in the PD-1 and PD-L1 groups are summarized in Figure 5. For the PD-1 group, among the 13 TRAEs listed, the most common five were elevated aspartate aminotransferase (27.6%), hypertension (25.9%), diarrhea (21.0%), proteinuria (20.3%), and decreased platelet count (20.1%). For the PD-L1 group, diarrhea (30.3%), elevated aspartate transaminase (20.2%), fatigue (19.7%), decreased appetite (19.2%), and hypertension (18.8%) were the commonest. The details for the other TRAEs are shown in Supplementary Table 5 (Supplemental Digital Content 4, http://links.lww.com/JS9/A904). Thus, for both the PD-1 and PD-L1 groups, the top five commonest TRAEs were diarrhea (25.1%), elevated aspartate aminotransferase (24.3%), hypertension (22.8%), elevated alanine aminotransferase (18.7%), and decreased appetite (15.7%). All these TRAEs were closely related to the gastrointestinal tract, skin, and the endocrine system.

## Discussion

This is a systematic review and meta-analysis on immunotherapy trials in treating patients with advanced HCC which were published from January 2002 to October 2022. Of 1392 articles on this topic, after using predetermined and strict quality assessment criteria, 12 studies (eight high-quality first-line RCTs and four second-line non-RCTs) with 5948 advanced HCC patients were included, and the clinico-pathological characteristics of patients which correlated with preferable treatment outcomes on OS, PFS, and ORR to immunotherapy were determined.

OS is the best treatment endpoint to be used in tumor clinical trials to assess systemic therapy. In this study, five trials were successful to reach to the predetermined end points of OS to show positive results to immunotherapy. For the first-line RCTs, our study showed that clinico-pathological characteristics of patients which correlated with preferable OS were no prior local therapy, EHS, AFP greater than or less than 400 ng/ml, viral hepatitis positivity, ECOG 1, MVI, MVI and/or EHS, BCLC C disease, age greater than or less than 65 years, and male. To better balance between the benefits and costs of immunotherapy, one study was removed based on the ESMO-MCBS (v1.1). Reanalysis of results did not alter from the above results obtained before the removal. Two important findings were found in the meta-analysis of OS in our study: the differences in OS between the viral hepatitis subgroups (especially in HBV subgroup) and the MVI and/or EHS subgroups were highly significant for interaction analysis, suggesting that the treatment responses of patients with viral hepatitis positivity and MVI and/or EHS to immunotherapy may be preferable than those without viral hepatitis and MVI and/or EHS. A systematic review and meta-analysis had previously published similar results in which the treatment outcomes to immunotherapy also suggested that ICI therapies were less effective in patients with nonviral etiologies than those with virus-associated HCC^32^. Further medical evidences clearly suggested that virus-related HCCs responded better to immunotherapy, while nonviral HCC, especially nonalcoholic steatohepatitis (NASH) related HCC showed decreased response to the self-invasion behavior of CD8+PD-1+T cells when compared to viral related HCC, indicating viral hepatitis shows a better treatment response to ICIs^33,34^. Meanwhile, a previously reported meta-analysis on HCC patients with EHS/MVI showed that Sorafenib improved OS^35^. In many RCTs which used Sorafenib as the control group, PD-1/PD-L1 inhibitors showed superior treatment outcomes than Sorafenib, with improved therapeutic efficacy in HCC patients with EHS/MVI. Overall, immunotherapy on OS showed an interesting trend according to our results that even patients with poor disease conditions would show improvement in OS.

PFS is a comprehensive endpoint used to capture death and disease progression. A longer PFS implies a longer period of better quality of life for patients. The results of COSMIC-312 met the predetermined primary endpoint of PFS in the first interim analysis, but failed to reach the endpoint of OS, indicating the importance of using PFS in addition to OS in oncological studies. PFS was provided in detail in the form of subgroups in four first-line RCTs. For treatment outcomes to immunotherapy, the clinico-pathological characteristics in patients which correlated with preferable PFS were BCLC B disease, viral hepatitis positivity, AFP less than 400 ng/ml, EHS/MVI, and prior local therapy. In the viral hepatitis group, when compared with the nonviral hepatitis group, viral hepatitis positivity may show a more significant improvement in PFS to immunotherapy (especially in the HBV subgroup). In three first-line RCTs (IMbrave-150, SHR-1210-III-310, COSMIC-312), patients with viral hepatitis, especially those with hepatitis B, responded better in PFS to immunotherapy, which is consistent with the results obtained in OS.

Based on the provided information, it can be observed that patients with BCLC C disease and AFP greater than or equal to 400 ng/ml may exhibit preferable OS with immunotherapy. Conversely, patients with BCLC B disease and AFP less than 400 ng/ml might experience preferable PFS response. This contrasting finding is similar to the results obtained from COSMIC-312, where the combination therapy of Cabozantinib and Atezolizumab demonstrated a significant extension in PFS among patients with advanced HCC compared to Sorafenib, without a corresponding significant improvement in OS. Multiple factors contribute to this outcome, including the longer follow-up duration for OS assessment and the variability in subsequent treatment options.

Contrary to OS and PFS, ORR stresses on results obtained in short-term treatment outcomes. Our meta-analysis showed the clinico-pathological characteristics of viral hepatitis positivity, AFP less than 400 ng/ml, ECOG 0, MVI, no EHS, and positive PD-L1 expression correlated with preferable ORR to immunotherapy. These clinico-pathological characteristics were basically similar to those obtained in PFS, suggesting that patients with better basic conditions may obtain better ORR to immunotherapy.

To sum up, for first-line immunotherapy, patients with viral hepatitis (especially HBV) may respond better to immunotherapy in OS and PFS. These results were consistent with the treatment outcomes obtained in IMbrave-150 and SHR-1210-III-310. In addition, five out of eight first-line RCTs showed that HCC patients with MVI and/or EHS showed improved responses to immunotherapy, indicating that the biological activity of tumor antigen-specific T cells exerts antitumor effects to inhibit tumor angiogenesis better in these patients^36–38^.

The problems of using Sorafenib in treating patients with advanced HCC are: drug resistance, and only around 30% of patients would respond to treatment; tolerance to Sorafenib, which usually occurs within 6 months of treatment^39^. All these make second-line treatments to become a necessity. In our analysis, patients with BCLC C disease may show preferable ORR to immunotherapy than BCLC B disease patients. However, as most reported second-line immunotherapy studies are phase II trials, further research is urgently needed. Our study showed treatment responses to PD-1/PD-L1 inhibitors were not up to that obtained by using Ramucrumab (REACH and REACH-2). Further research comparing PD-1/PD-L1 inhibitors and Ramucrumab is also needed^40,41^.

Toxicity arising from the use of PD-1/PD-L1 inhibitors has attracted great attention due to the increased popularity of using ICIs to treat HCC. In our study, the incidence of TRAEs in the PD-L1 group showed the PD-L1 antibody may have a relatively low toxicity. Although the difference after interaction did not achieve statistical significance, it also demonstrates to some extent the PD-1 inhibitors tend to exhibit a higher incidence of TRAEs. Additionally, other studies have provided evidence through theoretical and clinical data analysis, indicating that PD-L1 is associated with a lower incidence of toxicity compared to PD-1 inhibitors^42,43^. While PD-1 inhibitors act on PD-L1 and PD-L2 receptors of human T cells, the action site of PD-L1 depressors is only on PD-L1 of T cells and tumor cells, thus explaining why PD-L1 treatment has a lower toxicity with less collateral damage to the human body^44^. When PD-1/PD-L1 inhibitors are used jointly with VEGF antibody, although drug toxicities are superimposed, they in general are not overlapped^45^. When compared with the combined treatment with Bevacizumab in ORIENT-32 and IMbrave-150, the toxicity of treatment in using a single drug in Checkmate-459 and RATIONALE-301 was significantly less. The most common TRAEs of using PD-1 or PD-L1 inhibitors were diarrhea, increased aspartate aminotransferase and hypertension. These TRAEs should be looked for when PD-1 or PD-L1 inhibitors are used.

This meta-analysis has limitations. First, the number of patients included in the study was limited, and the analysis relied on data collected as a whole instead of individual patients. Second, this study focused on subgroup analysis, which is an exploratory study instead of real results of RCTs, but it can provide an important basis for the next step of conducting RCTs for appropriate subgroups of the population. With the current pace of development of immunotherapy, more first-line and second-line phase III RCTs on immunotherapy for advanced HCC are in progress. The outcomes of these studies on immunotherapy are expected to be published in the near future. All these studies, hopefully will promote immunotherapy of HCC to take a big step forward, especially in helping to figure out patient profiles which correlated with preferable treatment responses to immunotherapy.

## CONCLUSION

In conclusion, for first-line immunotherapy, subgroups of patients with viral hepatitis (especially HBV) and MVI and/or EHS may respond better in OS to immunotherapy. Patients with viral hepatitis would also respond better in PFS to immunotherapy. For second-line immunotherapy, patients with BCLC C disease may respond better in ORR to immunotherapy than BCLC B group. PD-1 inhibitors may have a higher probability to cause TRAEs compared to PD-L1 inhibitors. This systematic review and meta-analysis is the first pilot study to determine the key clinico-pathological characteristics of advanced HCC patients in predicting preferable treatment outcomes on OS, PFS, and ORR to immunotherapy.

## Ethical approval

This study did not require ethical approval.

## Consent

Not applicable.

## Sources of funding

This work was supported by the National Natural Science Foundation of China (81773140).

## Author contribution

Y.W.: conceptualization, data curation, methodology, formal analysis, software, visualization, writing – original draft; F.X.: conceptualization, methodology,visualization, writing – review and editing; J.W.: writing – review and editing; W.L.: visualization, writing – review and editing; Y.L.: methodology, writing – review and editing; Y.T. and Y.L.: formal analysis.

## Conflicts of interest disclosure

The authors declare that the research was conducted in the absence of any commercial or financial relationships that could be construed as a potential conflicts of interest.

## Research registration unique identifying number (UIN)

1. Name of registry: PROSPERO.
2. Registration ID: CRD42023425216.
3. Hyperlink to my specific registration: https://www.crd.york.ac.uk/prospero/display_record.php?ID=CRD42023425216.

## Guarantor

Feng Xia.

## Data availability statement

Results reported in the study underlie data of included clinic trials, and can be available to other researchers via a request: frankfxia@163.com.

## Provenance and peer review

Not commissioned, externally peer-reviewed.

## Supplementary Material
