# Supplementary material for: Clinico-characteristics of patients which correlated with preferable treatment outcomes in immunotherapy for advanced hepatocellular carcinoma: a systematic review and meta-analysis
Source: Int J Surg. 2023 Aug 17;109(11):3590–601. doi: 10.1097/JS9.0000000000000652 (PMC10651248; doi:10.1097/JS9.0000000000000652)
Supplement: Supplementary file 2 [file js9-109-3590-s002.docx]

Records identified from:

Databases (n =1392)

Other resources (n = 3)

Records removed *before screening*:

Duplicate records removed

(n = 450)

**Identification**

Records excluded

(n = 841)：

Not in line with HCC

Non immune related content

Non clinical trial

Records screened

(n = 945)

Reports excluded

(n = 92)：

Non phase III and phase II

No complete subgroup data record (OS、PFS、ORR)

Reports sought for retrieval

(n = 104)

**Screening**

Reports assessed for eligibility

(n = 12)

Studies included in review

(n = 12)

**Included**

*Consider, if feasible to do so, reporting the number of records identified from each database or register searched (rather than the total number across all databases/registers).

**If automation tools were used, indicate how many records were excluded by a human and how many were excluded by automation tools.

*From:*  Page MJ, McKenzie JE, Bossuyt PM, Boutron I, Hoffmann TC, Mulrow CD, et al. The PRISMA 2020 statement: an updated guideline for reporting systematic reviews. BMJ 2021;372:n71. doi: 10.1136/bmj.n71

For more information, visit: <http://www.prisma-statement.org/>
