# Supplementary material for: Clinico-characteristics of patients which correlated with preferable treatment outcomes in immunotherapy for advanced hepatocellular carcinoma: a systematic review and meta-analysis
Source: Int J Surg. 2023 Aug 17;109(11):3590–601. doi: 10.1097/JS9.0000000000000652 (PMC10651248; doi:10.1097/JS9.0000000000000652)
Supplement: Supplementary file 4 [file js9-109-3590-s004.docx]

**Clinico-characteristics of patients which correlated with preferable treatment outcomes in immunotherapy for advanced hepatocellular carcinoma: a systematic review and meta-analysis**

1. **SUPPLEMENTARY FIGURES**

- Figure S1. Publication bias analysis: funnel plot analysis of first-line RCTs(a); Egger’s test of first-line RCTs(b)
- Figure S2. Forest plot analysis of the subgroup of viral hepatitis to first-line immunotherapy
- Figure S3. OS-Forest plot analysis of each subgroup to first-line studies using targeted and immunotherapy
- Figure S4. OS-Forest plot analysis of each subgroup to first-line immunotherapy after ESMO-MCBS (v1.1) scoring
- Figure S5. Forest plot analysis of treatment-related adverse events in the PD-1 Group versus the PD-L1 Group.
- Figure S6. Risk assessment of bias in the included RCT studies

1. **SUPPLEMENTARY TABLES**

- Table S1. Detailed search strategies
- Table S2. Literature quality rating tables for first-line RCT articles
- Table S3. Literature quality rating tables for second-line non-RCT articles
- Table S4. Scores of RCT studies after ESMO-MCBS (v1.1) scoring
- Table S5. Treatment-related adverse events for all grades of the PD-1 group and the PD-L1 group

**3. SENSITIVITY ANALYSIS**

**1. SUPPLEMENTARY FIGURES**

**Figure S1. Publication bias analysis: funnel plot analysis of first-line RCTs(a); Egger’s test of first-line RCTs(b)**

**(a)**

**
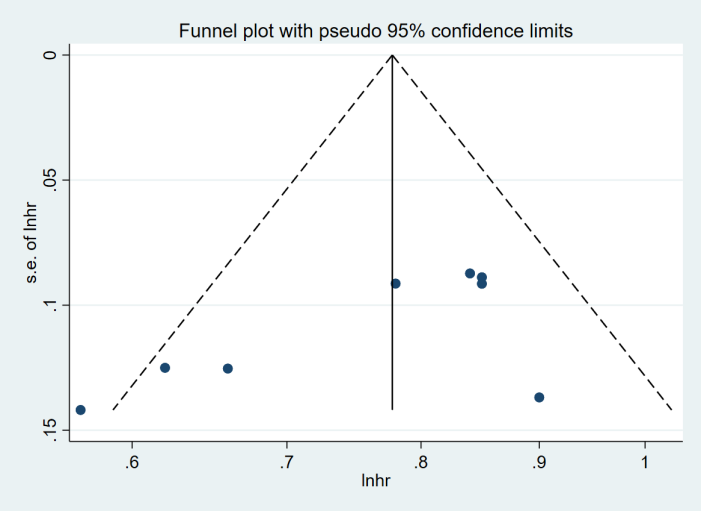
**

**(b)**


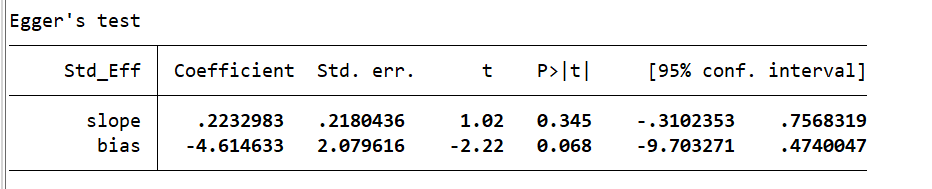


**Figure S1. Publication bias analysis: funnel plot analysis of first-line RCTs(a); Egger’s test of first-line RCTs(b).** a. The funnel plot included 8 RCT studies and showed a slight asymmetric. COSMIC-312 was outside the confidence interval. b. the Egger’s test showed that p=0.068 (>0.5), indicating that there was no publication bias in this study.

**Figure S2. Forest plot analysis of the subgroup of viral hepatitis to first-line immunotherapy**

**Figure S2. Forest plot analysis of the subgroup of viral hepatitis to first-line immunotherapy.** If I^2^ >50%, the random effects model was used, on the contrary, the fixed effects model was used. P<0.05 was defined as statistically significant. P value for interaction indicated whether there were significant differences in subgroups, and p<0.05 was defined as significant difference (the difference in OS and PFS in HBV subgroup are significant). HBV, Hepatitis B Virus; HCV, Hepatitis C Virus.

**Figure S3. OS-Forest plot analysis of each subgroup to first-line studies using targeted and immunotherapy**

**Figure S3. OS-Forest plot analysis of each subgroup to first-line studies using targeted and immunotherapy.** Viral hepatitis and MVI and/or EHS subgroups had significant difference in OS according to interaction analysis. In each subgroup, if I2 >50%, the random effects model was used, on the contrary, the fixed effects model was used. P<0.05 was defined as statistically significant. P value for interaction indicated whether there were significant differences in subgroups, and p<0.05 was defined as significant difference. Prior local therapy refers to surgery, ablation, liver transplant, radiotherapy and so on. CI, confidence interval; OS, overall survival; BCLC, Barcelona Clinic Liver Cancer; AFP, alpha-fetoprotein; ECOG, Eastern Cooperative Oncology Group; MVI, macrovascular invasion; EHS, extrahepatic spread. PD-L1, immune checkpoint inhibitors, programmed death-ligand 1.

**
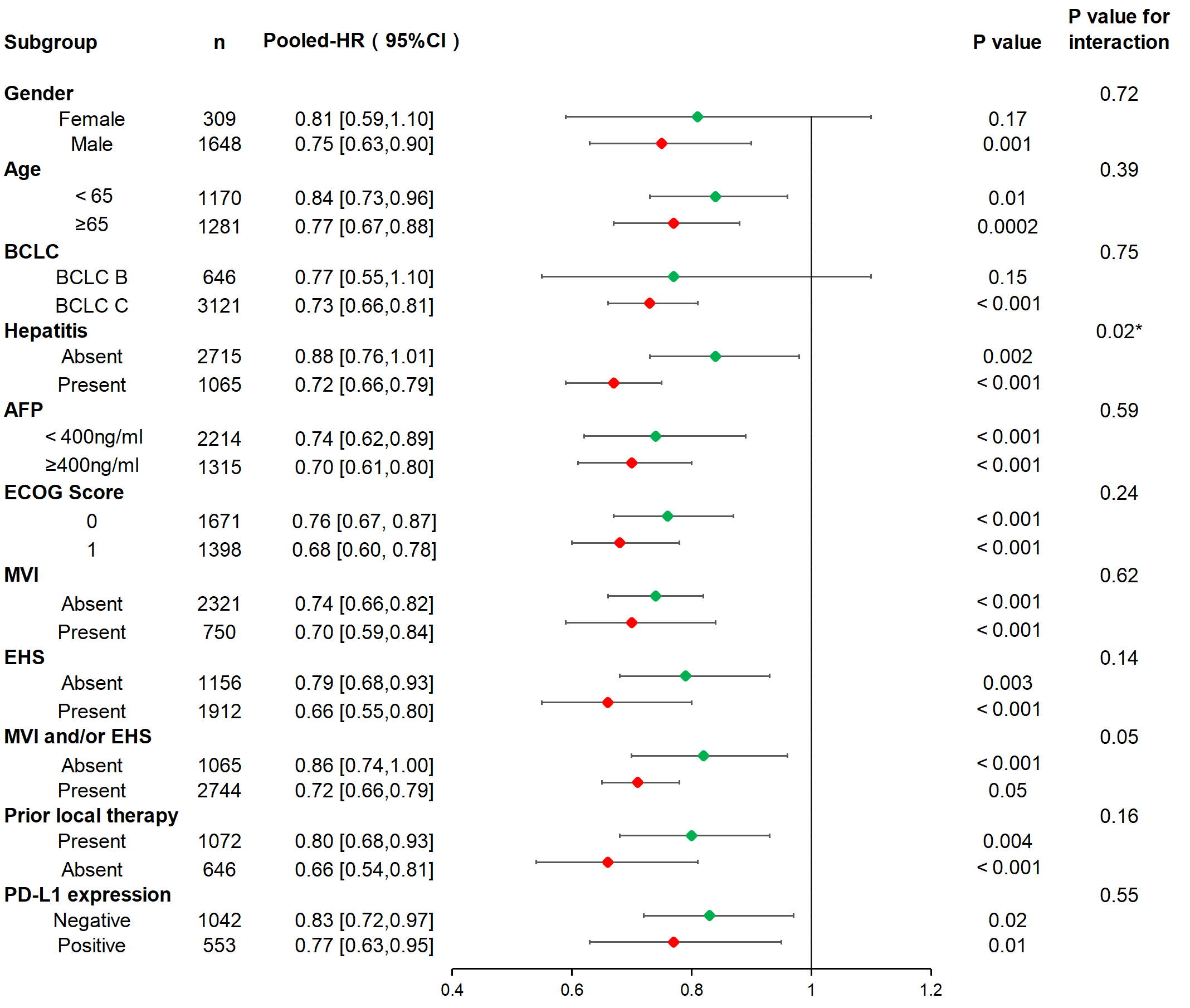
Figure S4. OS-Forest plot analysis of each subgroup to first-line immunotherapy after ESMO-MCBS (v1.1) scoring**

**Figure S4. OS-Forest plot analysis of each subgroup to first-line immunotherapy after ESMO-MCBS (v1.1) scoring.** In each subgroup, if I^2^ >50%, the random effects model was used, on the contrary, the fixed effects model was used. P<0.05 was defined as statistically significant. P value for interaction indicated whether there were significant differences in subgroups, and p<0.05 was defined as significant difference (the difference in OS in viral hepatitis group remained significant, in the MVI and/or EHS group it was just slightly significant). Prior local therapy refers to surgery, ablation, liver transplant, radiotherapy and so on. CI, confidence interval; OS, overall survival; BCLC, Barcelona Clinic Liver Cancer; AFP, alpha-fetoprotein; ECOG, Eastern Cooperative Oncology Group; MVI, macrovascular invasion; EHS, extrahepatic spread. PD-L1, immune checkpoint inhibitors, programmed death-ligand 1.

**Figure S5. Forest plot analysis of treatment-related adverse events in the PD-1 Group versus the PD-L1 Group.**

**Figure S5. Forest plot analysis of treatment-related adverse events in the PD-1 Group versus the PD-L1 Group.** P value for interaction indicated whether there were significant differences in subgroups, and p<0.05 was defined as significant difference. PD-1 equals to the PD-1 Group, and PD-L1 equals to the PD-L1 Group. AE, adverse event.

**Figure S6. Risk assessment of bias in the included RCT studies**

a. Risk of bias summary

**
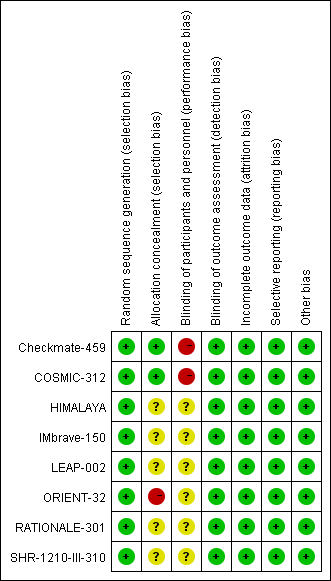
**

b. Risk of bias graph

**
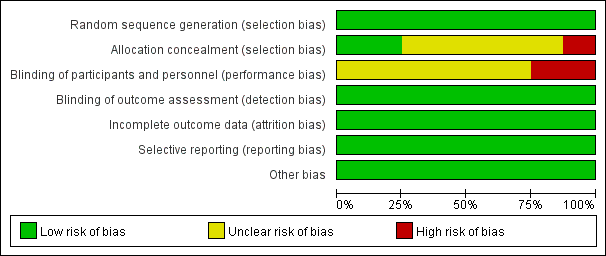
**

**2. SUPPLEMENTARY TABLES**

**Table S1. Detailed search strategies**

| Search | Database | Query | Results |
| --- | --- | --- | --- |
| 1 | PubMed  (Included in MEDLINE) | (((("carcinoma, hepatocellular"[MeSH Terms] OR "hepatocellular carcinoma"[Title/Abstract] OR "liver cancer"[Title/Abstract] OR "HCC"[Title/Abstract] OR "liver tumor"[Title/Abstract] OR "liver neoplasms"[Title/Abstract]) AND ("Immunotherapy"[MeSH Terms] OR "Immunotherapy"[Title/Abstract] OR "antibodies monoclonal"[Title/Abstract] OR "Mab"[Title/Abstract] OR "Atezolizumab"[Title/Abstract] OR "Sintilimab"[Title/Abstract] OR "Nivolumab" [Title/Abstract] OR "Pembrolizumab"[Title/Abstract] OR "Camrelizumab"[Title/Abstract] OR "Tremelimumab"[Title/Abstract])) NOT "Case Reports"[Publication Type]) NOT "Systematic Review"[Publication Type]) AND ((clinical trial[Filter]) AND (2002:2022[pdat])) | 204 |
| 2 | Web of Science | (TS=(carcinoma, hepatocellular OR Carcinomas, Hepatocellular OR Hepatocellular Carcinomas OR Liver Cell Carcinoma, Adult OR Liver Cancer, Adult OR Adult Liver Cancer OR Adult Liver Cancers OR Cancer, Adult Liver OR Cancers, Adult Liver OR Liver Cancers, Adult OR Liver Cell Carcinoma OR Carcinoma, Liver Cell OR Carcinomas, Liver Cell OR Cell Carcinoma, Liver OR Cell Carcinomas, Liver OR Liver Cell Carcinomas OR Hepatocellular Carcinoma OR Hepatoma OR Hepatomas) AND TS=( Immunotherapy OR Atezolizumab OR Sintilimab OR Nivolumab OR Pembrolizumab OR Camrelizumab OR Tremelimumab) AND TS=(randomized controlled trial OR controlled clinical trial)) AND (2002-2022 Filter) | 521 |
| 3 | Cochrane Library | (hepatocellular carcinoma or liver cancer or HCC or liver tumor or liver neoplasms):ti,ab,kw AND (Immunotherapy or antibodies monoclonal or Mab or Atezolizumab or Sintilimab or Nivolumab or Pembrolizumab or Camrelizumab or Tremelimumab):ti,ab,kw AND (randomized controlled trial OR controlled clinical trial) :ti,ab,kw AND (2002-2022 Filter) | 667 |

**Table S2. Literature quality rating tables for first-line RCT articles**

| **Questions**  **Studies** | The generation of random sequences | Randomized hiding | Blind method | Withdrawal and loss of follow-up | score |
| --- | --- | --- | --- | --- | --- |
| COSMIC-312 | 2 | 2 | 2 | 1 | 7 |
| IMbrave-150 | 2 | 2 | 2 | 1 | 7 |
| ORIENT-32 | 2 | 2 | 2 | 1 | 7 |
| CheckMate-459 | 2 | 1 | 2 | 1 | 6 |
| HIMALAYA | 1 | 1 | 2 | 1 | 5 |
| LEAP-002 | 2 | 2 | 2 | 1 | 7 |
| RATIONALE-301 | 2 | 2 | 2 | 1 | 7 |
| SHR-1210-III-310 | 2 | 2 | 2 | 1 | 7 |

**Table S3. Literature quality rating tables for second-line non-RCT articles**

| **Questions**  **Studies** | The purpose of the research is clearly given | Continuity of inclusion of patients | Collection of expected data | The endpoints reflect the objective of the study | The objectivity of the end point index evaluation | Whether the follow-up time was sufficient | The loss of follow-up rate is less than 5% | Whether the sample size was estimated | score |
| --- | --- | --- | --- | --- | --- | --- | --- | --- | --- |
| KEYNOTE-224 | 2 | 2 | 2 | 2 | 1 | 2 | 2 | 1 | 14 |
| CheckMate-040 | 2 | 2 | 2 | 2 | 2 | 2 | 2 | 1 | 15 |
| RATIONALE-208 | 2 | 2 | 2 | 2 | 0 | 2 | 2 | 1 | 13 |
| RESCUE | 2 | 2 | 2 | 2 | 1 | 2 | 2 | 1 | 14 |

**Table S4. Scores of RCT studies after ESMO-MCBS (v1.1) scoring**

| **Trial** | **Treatment** | **OS** | | | | **PFS** | | | | **QoL*** | **Toxicity^#^** | **ESMO**  **Score** |
| --- | --- | --- | --- | --- | --- | --- | --- | --- | --- | --- | --- | --- |
|  |  | **Median**  **mo** | **Gain** | **HR**  **(95% CI)** | **P**  **value** | **Median**  **mo** | **Gain** | **HR**  **(95% CI)** | **P**  **value** |  |  |  |
| COSMIC-312  2022 | Atezolizumab  +Cabozantinib | 15.4/  15.5 | - | 0.90  (0.69-1.18) | 0.44 | 6.8/4.2 | 2.6 | 0.63  (0.44-0.91) | 0.0012 | - | - | **3** |
| IMbrave-150  2021 | Atezolizumab  +Bevacizumab | 19.2/  13.4 | 5.8 | 0.66  (0.52-0.85) | <0.001 | 6.9/4.3 | 2.6 | 0.65  (0.53-0.81) | < 0.001 | **-** | **-** | **4** |
| ORIENT-32  2021 | Sintilimab +Bevacizumab | Na/  10.4 | - | 0.57  (0.43-0.75) | <0.0001 | 4.6/2.8 | 1.8 | 0.56  (0.46-0.70) | <0.0001 | Improved | **-** | **4** |
| CheckMate-459  2021 | Nivolumab | 16.4/  14.7 | 1.7 | 0·85  (0.72–1.02) | 0.075 | 3.7/3.8 | - | 0.98  (0.82-1.18) | **-** | Improved | Reduced | **3** |
| HIMALAYA  2022 | Tremelimumab +Durvalumab | 16.43/  13.77 | 2.66 | 0.78  (0.65-0.93) | 0.0035 | 3.78/4.07 | - | 0.90  (0.77-1.05) | **-** | Improved | **-** | **3** |
| LEAP-002  2022 | Pembrolizumab  + Lenvatinib | 21.2/  19.0 | 2.2 | 0.840  (0.708-0.997) | 0.0227 | 8.2/8.0 | 0.2 | 0.867  (0.734-1.024) | 0.0466 | **-** | **-** | **2** |
| RATIONALE  -301  2022 | Tislelizumab | 15.9/  14.1 | 1.8 | 0.85  (0.712-1.019) | 0.0398 | 2.1/3.4 | - | 1.1  (0.92-1.33) | **-** | **-** | Reduced | **3** |
| SHR-1210-III  -310  2022 | Camrelizumab  +Rivoccranib | 22.1/  15.2 | 6.9 | 0.62  (0.49-0.80) | <0.0001 | 5.6/3.7 | 1.9 | 0.52  (0.41-0.65) | <0.0001 | **-** | **-** | **4** |

**Table S4. Scores of RCT studies after ESMO-MCBS (v1.1) scoring**. Gain = the difference between the median OS/PFS of the control arm and treatment arm. When calculating ESMO score, OS was the first priority. If OS was not available, PFS was used for calculation. *The duration of overall health deterioration or quality of life of patients in the study group was significantly better than that in the control group, which was defined as improvement. # Overall toxicity was significantly reduced in the study group compared to the control group, defined as reduced toxicity. CI, confidence interval; OS, overall survival; PFS, progression free survival; QoL, quality of life; ESMO, European Society for Medical Oncology; mo, month; Na, not available.

| Specific TRAE | PD-1 group | | | | | Specific TRAE | PD-L1 group | | | |
| --- | --- | --- | --- | --- | --- | --- | --- | --- | --- | --- |
|  | ORIENT-32  (n=380) | CheckMate-459  (n=367) | LEAP-002  (n=395) | RATIONALE  -301  (n=338) | Portion  （%） |  | IMbrave-150  (n=329) | COSMIC-312  (n=429) | HIMALAYA  (n=388) | Portion  （%） |
| AST↑ | 135(36) | 39(11) | 107(27) | 127(37) | 27.6 | Diarrhea | 36(11) | 208(48) | 103 (27) | 30.3 |
| Hypertension | 121(32) | 3(1) | 238(60) | 21(6) | 25.9 | AST↑ | 54(16) | 129(30) | 48 (12) | 20.2 |
| Diarrhea | 55(14) | 31(8) | 187(47) | 38(11) | 21.0 | Fatigue | 54(16) | 106(25) | 66 (17) | 19.7 |
| Proteinuria | 160(42) | - | 141(36) | - | 20.3 | Appetite↓ | 38(12) | 116(27) | 66 (17) | 19.2 |
| Platelets↓ | 155(41) | - | 95(24) | 48(14) | 20.1 | Hypertension | 93(28) | 100(23) | 23 (6) | 18.8 |
| ALT↑ | 99(26) | - | 92(23) | 96(28) | 19.4 | ALT↑ | 40(12) | 127(30) | 36 (9) | 17.7 |
| Bilirubin↑ | 112(29) | - | 88(22) | 75(22) | 18.6 | PPE Syndrome | 5(2) | 183(43) | 3 (1) | 16.7 |
| Hypothyroidism | 53(14) | - | 162(41) | - | 14.5 | Rash | 32(10) | 61(14) | 87 (22) | 15.7 |
| Appetite↓ | 43(11) | 22(6) | 127(32) | - | 13.0 | Pruritus | 47(14) | 43(10) | 89 (23) | 15.6 |
| Fatigue | - | 57(15) | 116(29) | - | 11.7 | Hypothyroidism | 33(10) | 89 (21) | 47 (12) | 14.7 |
| PPE Syndrome | 1(＜1) | 4(1) | 143(36) | 1(＜1) | 10.1 | Proteinuria | 95(29) | - | - | 8.3 |
| Weight↓ | 57(15) | 4(1) | 88(22) | - | 10.1 | Platelets↓ | 34(10) | 52(12) | - | 7.5 |
| Pruritus | 39(10) | 46(12) | - | - | 5.7 | Bilirubin↑ | - | 56(13) | 20 (5) | 6.6 |
| Rash | 26(7) | 39(11) | - | - | 4.4 | Weight↓ | - | 73(17) | - | 6.4 |

**Table S5. Treatment-related adverse events for all grades of the PD-1 group and the PD-L1 group.**

**Table S5. Treatment-related adverse events for all grades of the PD-1 group and the PD-L1 group**. Data are n (%). PPE syndrome, Palmar-plantar erythrodysesthesia ALT, alanine aminotransferase; AST, aspartate aminotransferase; ↓ for decrease; ↑ for increase.

**3. SENSITIVITY ANALYSIS**

*1.OS*

| AFP(＜400) | AFP(≥400) |
| --- | --- |
| **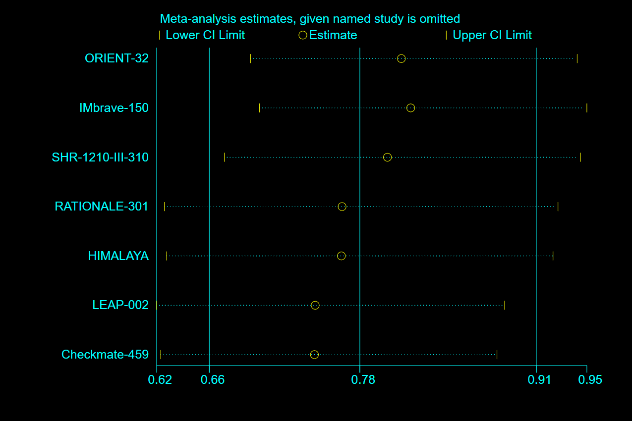** | **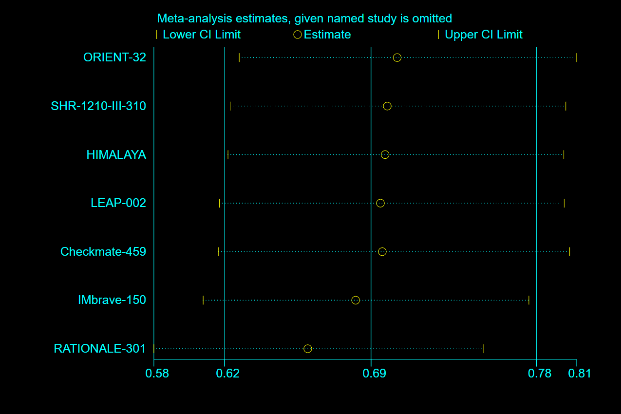** |
| Age(＜65)—unstable | Age(≥65) |
| **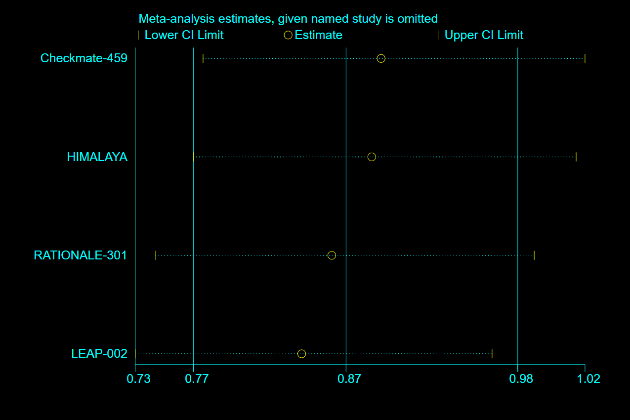** | **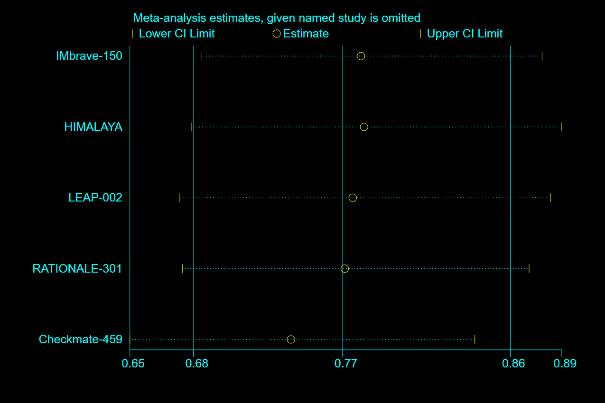** |
| BCLC B—unstable | BCLC C |
| 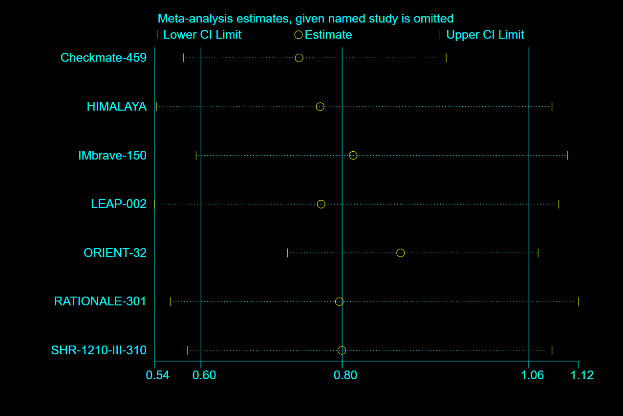 | 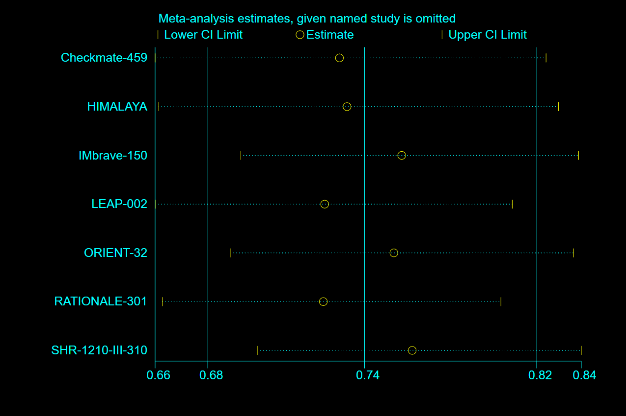 |
| ECOG 0 | ECOG 1 |
| **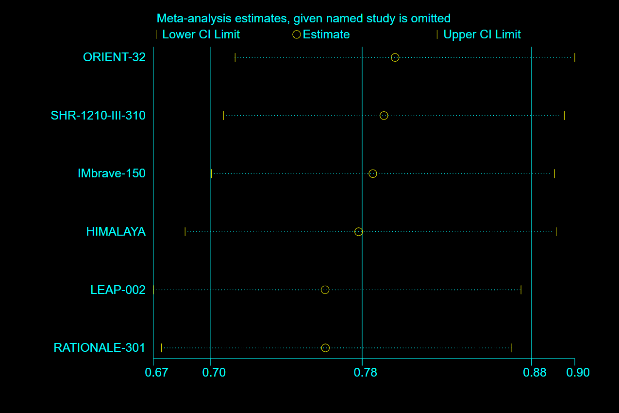** | **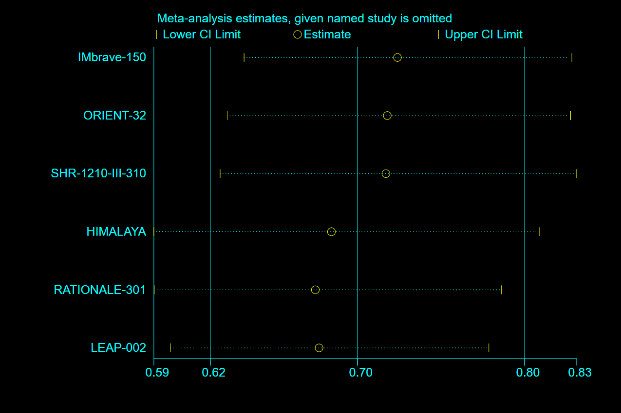** |
| EHS(YES) | EHS(NO)—unstable |
| **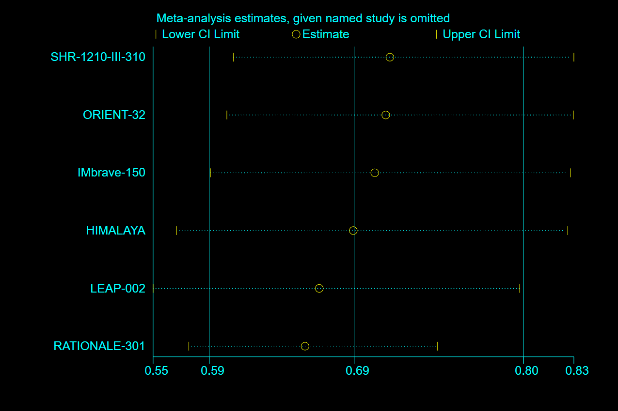** | **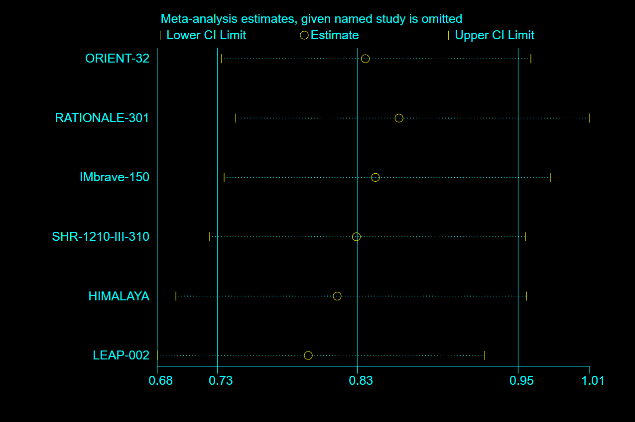** |
| Gender(male) | Gender(female) |
| **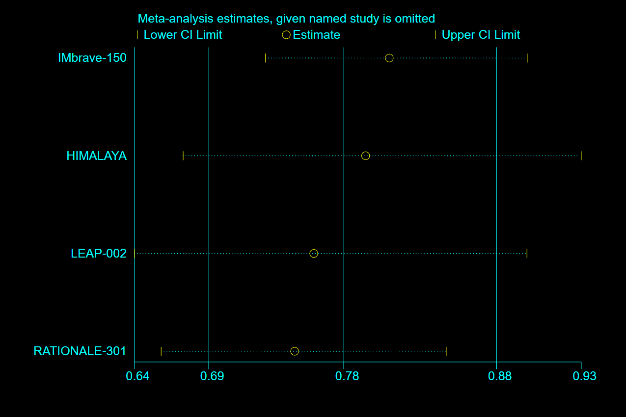** | **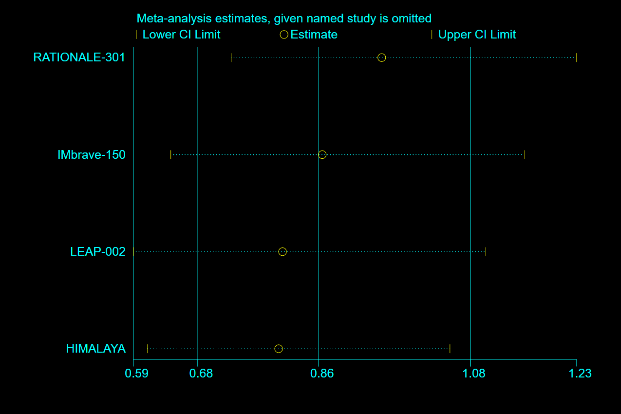** |
| MVI(YES) | MVI(NO) |
| **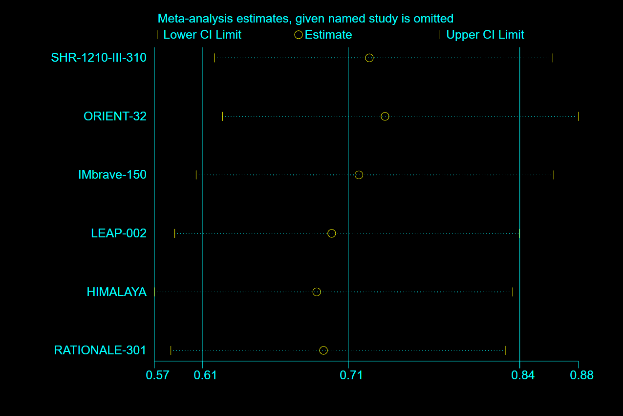** | **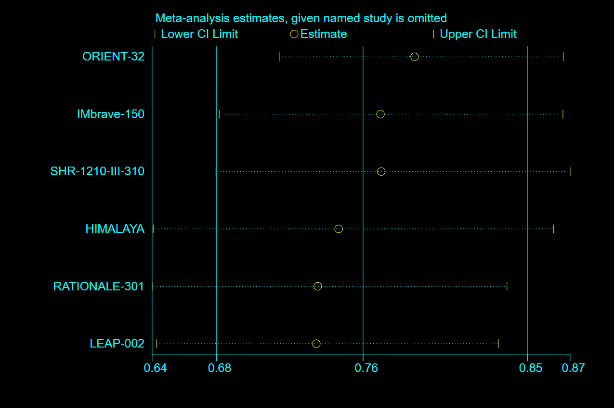** |
| MVI and/or EHS(YES) | MVI and/or EHS(NO) |
| **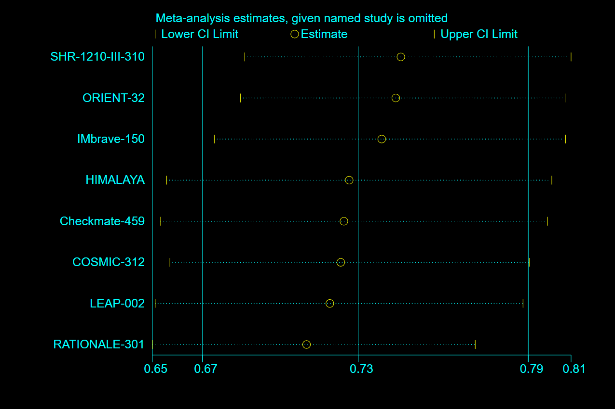** | **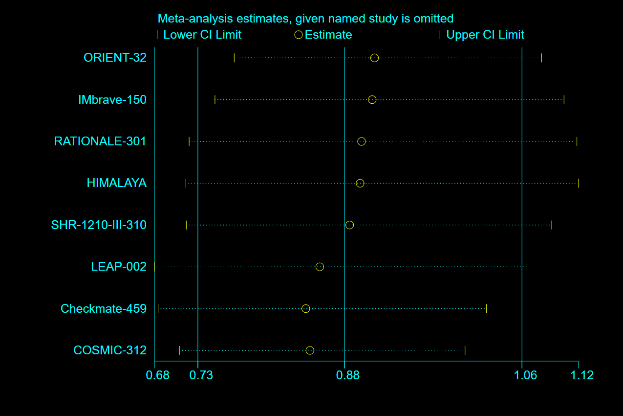** |
| PD-L1 expression(positive)—unstable | PD-L1 expression(negative)—unstable |
| 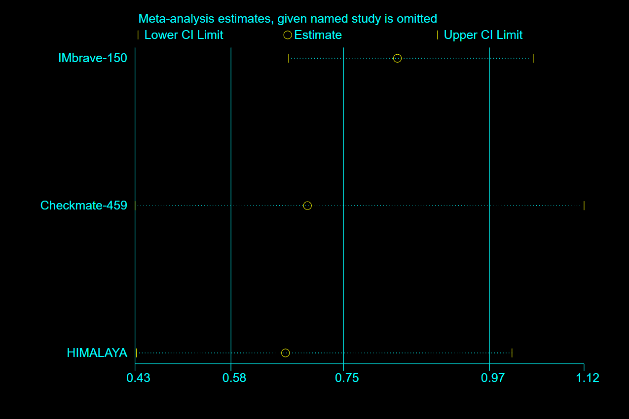 | **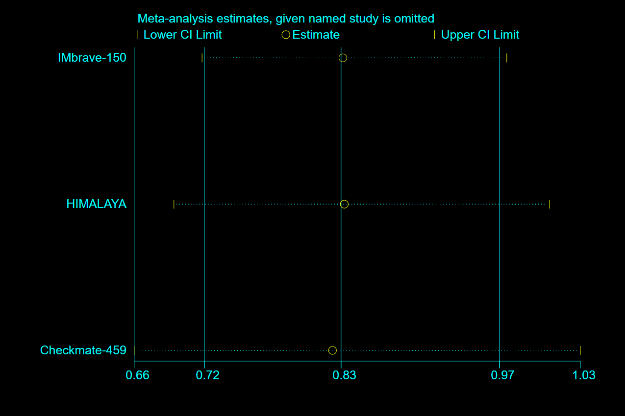** |
| prior local therapy (YES) | prior local therapy (NO) |
| 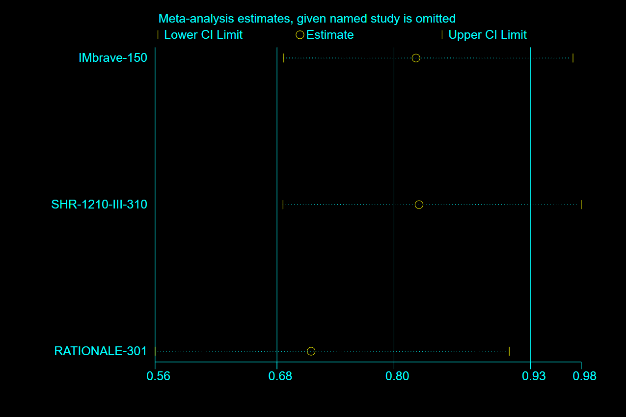 | **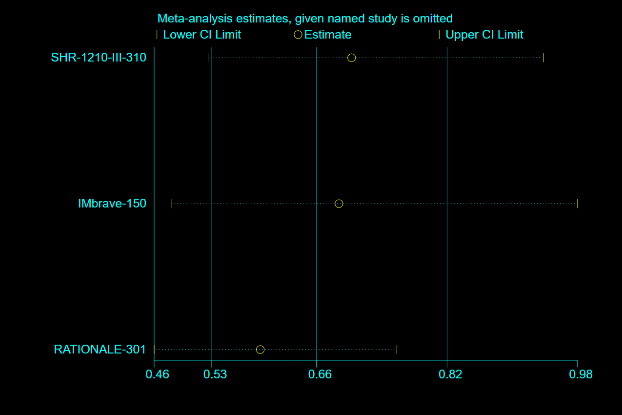** |
| viral(positive) | viral(negative)—unstable |
| 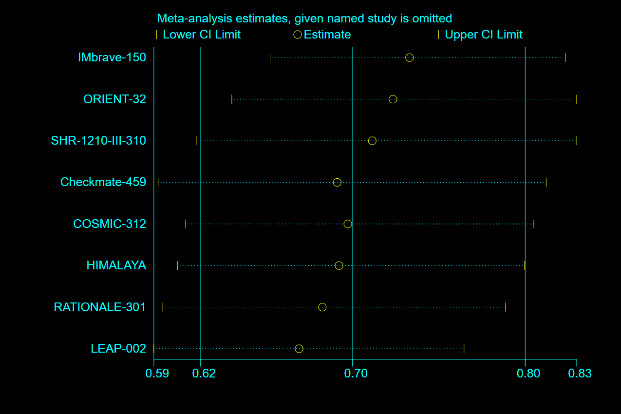 | **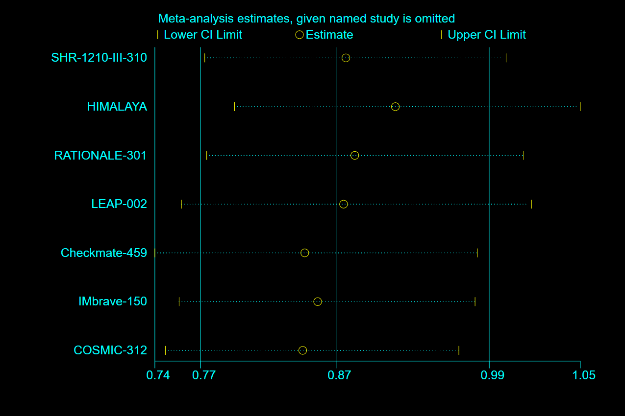** |

*2.PFS*

| AFP(＜400) | AFP(≥400)—unstable |
| --- | --- |
| **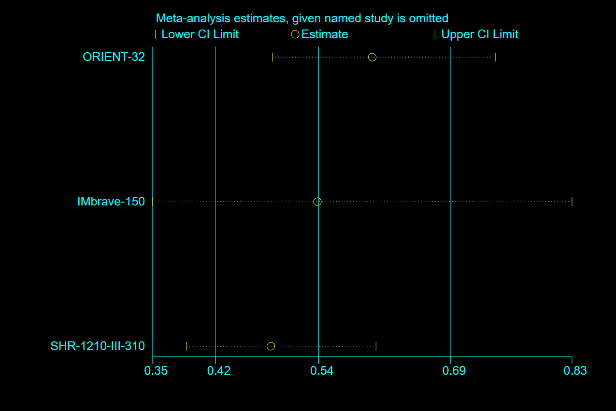** | **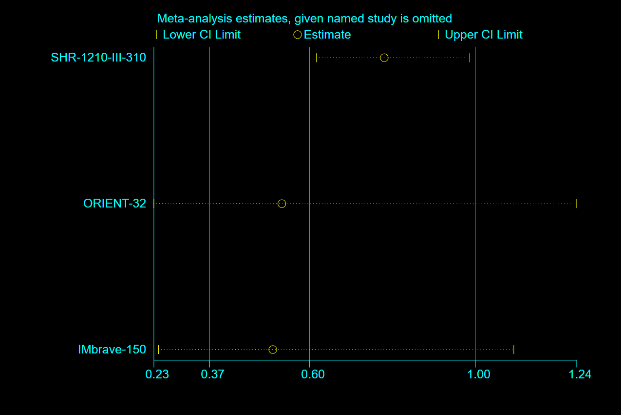** |
| BCLC B | BCLC C |
| **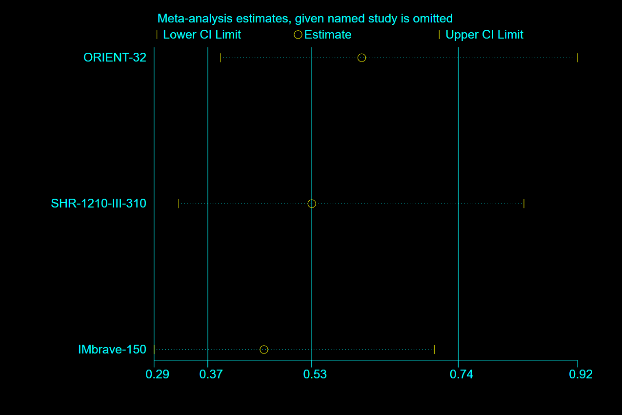** | **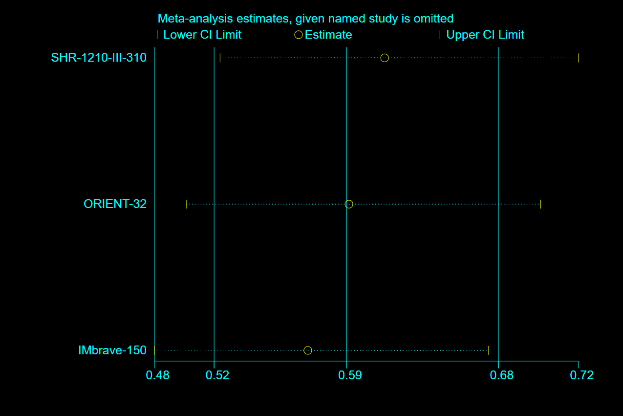** |
| ECOG 0 | ECOG 1 |
| **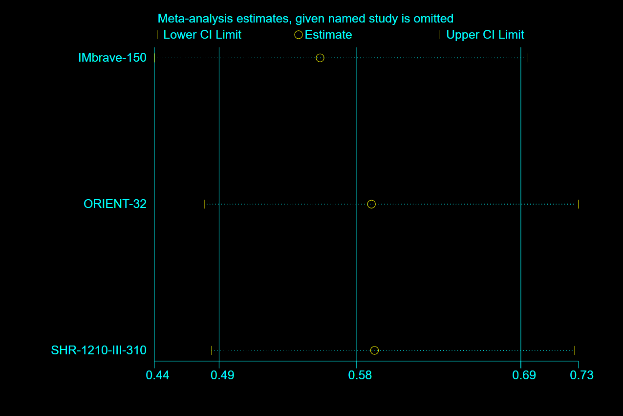** | **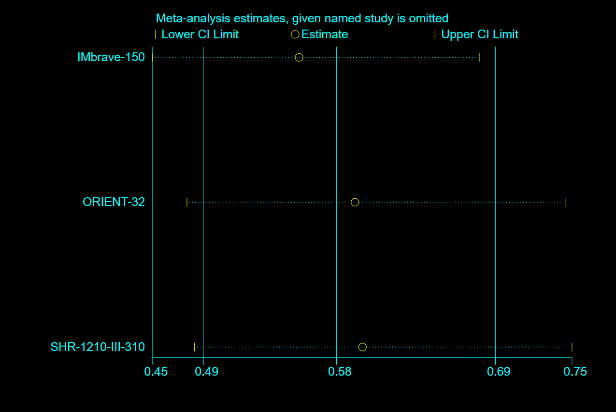** |
| EHS(YES) | EHS(NO) |
| 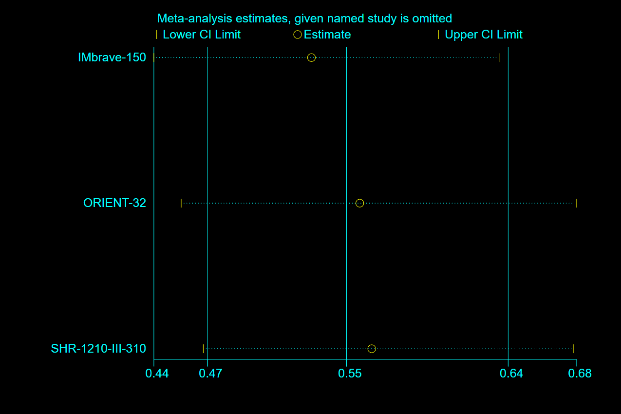 | **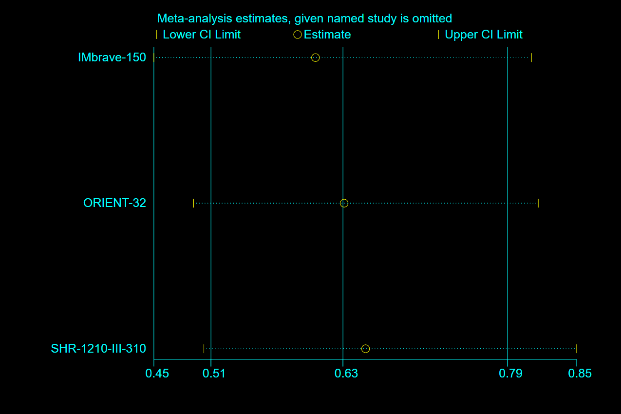** |
| MVI(YES) | MVI(NO) |
| 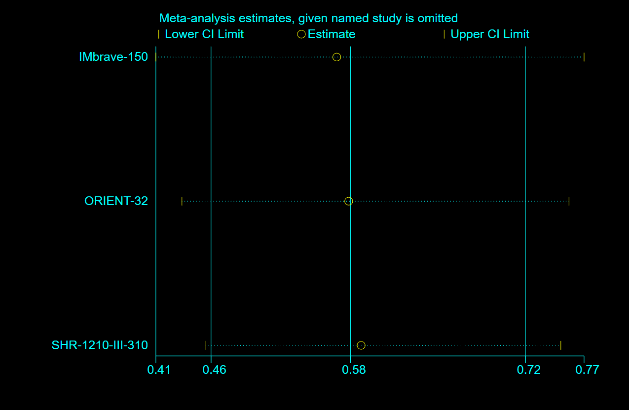 | 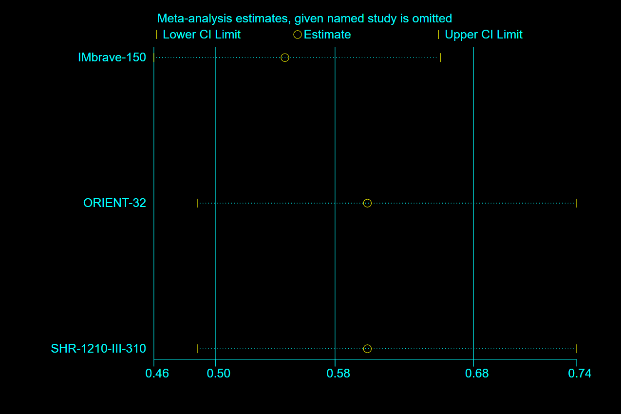 |
| MVI and/or EHS(YES) | MVI and/or EHS(NO)—unstable |
| 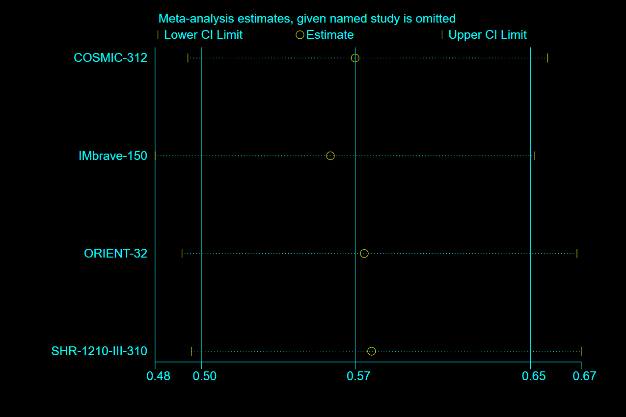 | 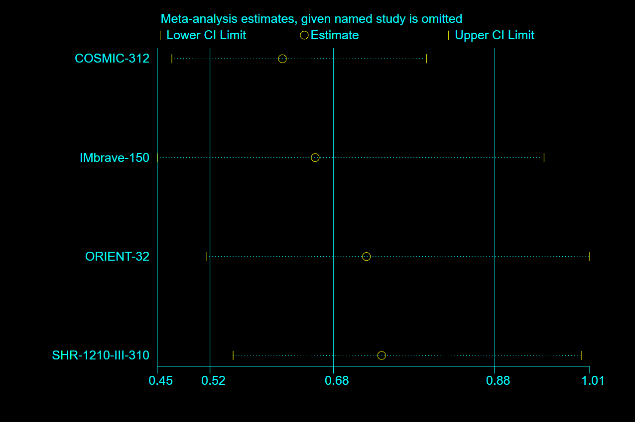 |
| viral(positive) | viral(negative)—unstable |
| 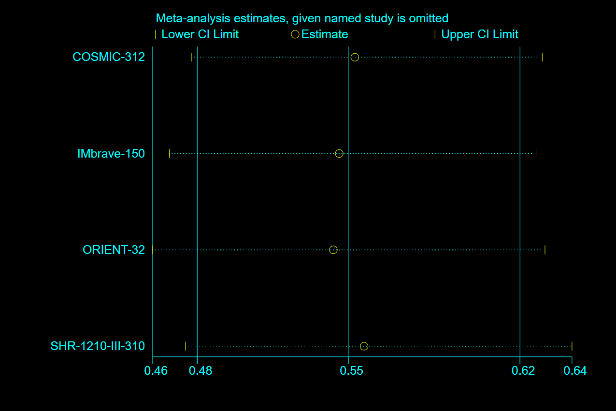 | 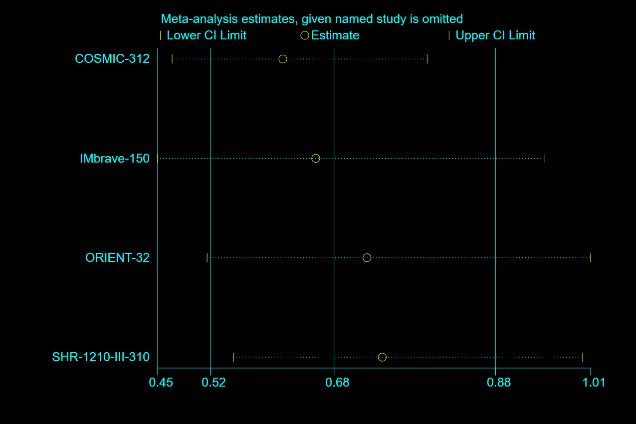 |
